# Non-Invasive Imaging for Subclinical Coronary Atherosclerosis in Patients with Peripheral Artery Disease

**DOI:** 10.1007/s11883-014-0415-3

**Published:** 2014-04-02

**Authors:** Rasmus Sejersten Ripa, Andreas Kjaer, Birger Hesse

**Affiliations:** 1 Department of Clinical Physiology, Nuclear Medicine and PET, KF-4012 Rigshospitalet University Hospital, Blegdamsvej 9, DK-2100 Copenhagen, Denmark; 2 Cluster for Molecular Imaging, Faculty of Health and Medical Sciences, University of Copenhagen, Copenhagen, Denmark

**Keywords:** Atherosclerosis, Imaging, Peripheral artery disease

## Abstract

Patients with peripheral artery disease are at high risk of coronary artery disease. An increasing number of studies show that a large proportion of patients with peripheral artery disease have significant coronary atherosclerosis, even in the absence of symptoms. Although the reported prevalence of subclinical coronary artery disease varies widely in patients with peripheral artery disease, it could include more than half of patients. No consensus exists to date on either the rationale for screening patients with peripheral artery disease for coronary atherosclerosis or the optimal algorithm and method for screening. An increasing number of imaging modalities are emerging that allow improved in vivo non-invasive characterization of atherosclerotic plaques. These novel imaging methods may lead to early detection of high-risk vulnerable plaques, enabling clinicians to improve risk stratification of patients with peripheral artery disease, and thus paving the way for individualized therapy.

## Introduction

Atherosclerosis is a systemic degenerative inflammatory vascular disease and the primary underlying cause of coronary heart disease. Atherosclerosis develops over decades, with a long subclinical period. About half of all patients who die from coronary heart disease have no prior diagnosis or symptoms of cardiac disease [1]. Despite this fact, screening asymptomatic adults for cardiovascular risk by imaging is considered inappropriate in most cases by current guidelines [2].

Clinical risk stratification of asymptomatic patients is typically performed using global risk scores such as the Framingham risk score or HeartScore, rendering a 10-year risk of cardiovascular death on the basis of “classic” cardiovascular risk factors. In addition to the clinical risk scores, a number of medical conditions indicate a cardiovascular risk similar to that of patients with established coronary heart disease [3••, 4]. These coronary heart disease risk equivalents are conditions such as diabetes and peripheral artery diseases (PAD), including lower limb arterial disease, abdominal aortic aneurism, and carotid artery disease. Given the high cardiac mortality and morbidity, early detection and treatment of subclinical coronary heart disease could potentially improve prognosis in patients with PAD.

This article will address the use of imaging methods for identification of subclinical coronary artery disease in patients with known PAD and will discuss the indications for imaging for subclinical coronary artery disease, both with regard to general screening and in the subgroup of patients with PAD who are referred for major surgery.

## Prevalence of Subclinical Coronary Artery Disease in Patients with PAD

More than 30 years ago, Hertzer et al. established that patients undergoing surgical management of PAD had a high prevalence of significant coronary artery disease and that a large proportion of the patients were asymptomatic [5]. It is now well documented that patients with atherosclerosis in major peripheral arteries also have a high risk of myocardial infarction [6].

A positive association between carotid and coronary atherosclerosis has been recognized for decades [7]. In the AMISTAD study of 405 patients with acute cerebral infarction [8], 85% who underwent coronary angiography had no known coronary artery disease. Coronary plaques were found in 62% and coronary stenoses (≥50%) in 26% of the asymptomatic patients. In addition, the burden of systemic atherosclerosis was determined by ultrasound examination of the carotid arteries, the aorta, and the femoral artery. After adjustment for traditional risk factors, the presence of coronary plaques was significantly associated with the presence of plaques in the carotid and femoral arteries. As was previously suggested by the REACH registry [9], polyvascular disease increased the risk of coronary plaques. In a recent report on clinical follow-up from the AMISTAD study [10•] both the presence and extent of coronary atherosclerosis were found to predict major vascular events in the 2-year follow-up period.

The prevalence of subclinical coronary artery disease reported in the AMISTAD study is supported by both the PRECORIS study [11] and autopsy studies following fatal stroke [12]. However, a limitation present in both AMISTAD and PRECORIS was the fact that patients were stratified according to known coronary disease (acute coronary syndrome, myocardial infarction, or revascularization), and thus it must be considered that some of these patients could be symptomatic (e.g., having mild angina or angina equivalents) though undiagnosed. If this is the case, one would expect that the prevalence of true subclinical coronary disease is overstated in these trials. Conversely, in a study of 420 patients with significant carotid stenosis, Hofmann et al. [13] found that approximately 60% of patients without cardiac symptoms had significant coronary stenoses as demonstrated by coronary angiography. It can be speculated that this observed difference in subclinical coronary artery disease is attributable to the differences in the cohorts studied (stroke vs. significant carotid stenosis).

A recent review of the literature including 1,277 patients reported that 50% of patients with abdominal aortic aneurisms had significant coronary artery disease [14]. This number should be regarded with caution, however, as the studies are heterogeneous with regard to inclusion criteria and methods and span more than four decades. Most of the studies included patients both with and without symptoms of myocardial ischemia. Only two studies specified the prevalence of true subclinical coronary artery disease in patients with abdominal aortic aneurism. Quigley and colleagues reported that 33% of 102 asymptomatic patients scheduled for surgical repair and screened by myocardial scintigraphy and/or by invasive angiography were found to have significant coronary artery disease [15]. Similarly, Kioka et al. found that 36% of asymptomatic patients with PAD had significant coronary stenosis (≥75%) by coronary angiography [16].

Patients with lower limb arterial disease are at increased risk of mortality from cardiovascular and coronary heart disease, even if PAD is asymptomatic [17]. Only a few studies have investigated subclinical coronary disease in patients with lower limb PAD. Her et al. performed coronary angiography prior to peripheral vascular surgery in 82 patients without symptoms of coronary heart disease and found that 70% had coronary atherosclerosis and 33% had ≥70% coronary stenosis [18]. In comparison, Hirose et al. performed stress-rest myocardial perfusion imaging in 183 patients with lower limb PAD and found significant presence of ischemia in 55% of the patients [19]. As some patients were reported to have angina pectoris, a direct comparison of the two studies is difficult. One very recent study included 200 patients with symptomatic lower limb PAD but without angina, dyspnea, or known coronary artery disease [20••]. Invasive angiography was performed in all patients and showed coronary atherosclerosis in 68%, with significant (≥70%) coronary stenosis in 55%.

In summary, the reported prevalence of subclinical coronary artery disease in patients with PAD varies widely (Tables 1). This may be attributable, at least in part, to a number of methodological differences. First, the definition of coronary symptoms is variable. For example, should dyspnea, syncope, or an abnormal ECG be designated as coronary symptoms? In addition, lower limb arterial disease may mask coronary ischemia as a result of functional limitations from the PAD. Second, the prevalence seems to vary according to the location of the PAD. Third, the accuracy of the screening method for coronary artery disease will affect the prevalence. Despite differences in reported prevalence, it is clear from all reported series that patients with PAD have a significantly increased prevalence of subclinical coronary artery disease and a high risk of subsequent complications.

**Table 1:** Prevalence of subclinical coronary artery disease in patients with symptomatic peripheral artery disease

Study	Year	Diagnostic modality	No. of patients	Kind of PAD	Prevalence of coronary stenoses	Symptoms of coronary artery disease
Hertzer et al.	1985	Coronary angiography	506	Carotid	16%	No
Amarenco et al.	2011	Coronary angiography	405	Stroke	26%	No known CAD
Calvet et al.	2010	CCTA	274	Stroke	18%	No known CAD
Hofmann et al.	2005	Coronary angiography	420	Carotid	60%	No
Hur et al.	1972-2012	Coronary angiography	1277	AAA	50%	Both symptomatic and asymptomatic
Quigley FG et al.	1999	Tl-201 SPECT	102	AAA	33%	No
Kioka et al.	2002	Coronary angiography	94	AAA	36%	No
Her et al.	2008	Coronary angiography	82	LEAD	33%	No
Hirose et al.	2009	Tl-201 SPECT	183	LEAD	55%	Both symptomatic and asymptomatic
Marsico et al.	2013	Coronary angiography	200	LEAD	55%	No

AAA=abdominal aortic aneurism, CCTA=coronary computed tomography angiography, CAD=coronary artery disease, LEAD=lower extremity arterial disease, PAD=peripheral artery disease, SPECT=single photon emission tomography

## Rationale for Screening for Subclinical Coronary Artery Disease in PAD

Screening for coronary artery disease (CAD) in patients with PAD remains a controversial subject, both in the general PAD population and for selected subgroups, and particularly in PAD patients referred for major surgery.

In patients with PAD who are referred for major surgery, whether for the PAD itself or for other diseases, the risk of postoperative cardiac morbidity and mortality is significantly higher, and preoperative screening for significant CAD is often recommended [21]. However, there is little evidence that supports screening for coronary disease and possible interventions as a means of reducing cardiac complications [21].

One recent study included 208 patients with PAD scheduled for major elective vascular surgery with a revised cardiac risk index ≥2 [22]. Patients were randomized to coronary angiography followed by extensive revascularization or to coronary angiography based on the result of ischemia testing (stress-echocardiography or stress-thallium scintigraphy). Revascularization was performed in 61 patients in the first group and 42 in the second group. On follow-up, patients randomized to routine coronary angiography showed better survival and freedom from death/cardiovascular events [22]. Unfortunately, this study did not specify whether any of these patients were without symptoms of myocardial ischemia. Additionally, the study compared two different imaging-derived strategies only. It would have been of interest also to compare to a group treated conservatively with only medication and lifestyle modification but without revascularization.

In the CARP trial, McFalls et al. had determined that revascularization before vascular surgery did not alter long-term outcome when compared to patients randomized to no revascularization [23]. This study included a highly selected cohort of PAD patients with stable cardiac symptoms and coronary angiography showing a significant stenosis. The results supported the contention that many acute cardiovascular events originate from stenoses that are without hemodynamic significance. A new analysis from the CARP trial further demonstrated that only patients with unprotected left main coronary artery disease had a prognostic benefit from preoperative coronary artery revascularization [24].

Patients with stroke are known to have high cardiovascular risk, even with optimal secondary therapy [10•]. One trial investigated the use of systematic preoperative coronary angiography, followed by percutaneous coronary intervention, before carotid endarterectomy in patients without clinical evidence of CAD [25]. All patients (*n* = 426) were without any clinical sign or history of ischemic heart disease. Patients were randomized to 1) angiography and percutaneous coronary intervention if a significant stenosis was detected or 2) no angiography prior to carotid endarterectomy. Systematic angiography was shown to significantly reduce the incidence of postoperative myocardial events. The 30-day survival and stroke rates were equal in the two groups [25]. Limitations of the study included few clinical endpoints and a short follow-up period. In addition, optimal postoperative medical treatment was not part of the study protocol.

If a significant CAD is demonstrated in the general population of PAD patients without coronary symptoms, should it then lead to medical prophylactic intervention or to coronary revascularization? In fact, very few studies have addressed the prognostic impact of image-guided therapy in this setting. Many will advocate that all patients with severe PAD should already be treated to maximal secondary prevention, and that imaging of the coronary arteries should not alter this treatment, and it is therefore not relevant. Others will advocate that significant coronary artery disease should lead to invasive treatment with percutaneous coronary intervention or bypass surgery in these asymptomatic but high-risk patients. A randomized controlled investigation is sorely needed to address these questions.

In summary, numerous studies have identified severe subclinical CAD in a substantial proportion of patients with PAD, but a beneficial effect of general screening for CAD by either noninvasive or invasive imaging on occurrence of coronary events has yet to be determined. Large prospective randomized trials are needed in order to provide clarity. As a preoperative investigation before intermediate or high-risk cardiac surgery, limited data suggest a beneficial effect of preoperative imaging of the coronary vessels or myocardial perfusion distribution.

From a theoretical risk reduction perspective, a potential benefit in both the general PAD patient population and as a preoperative coronary imaging study of subclinical CAD may be the identification of vulnerable plaques that are prone to rupture or to cause thrombus that may subsequently lead to myocardial infarction. Conceptually, this can be accomplished by imaging the plaque, the anatomy of the coronary vessel, or ischemia.

## Non-Invasive Imaging of the Coronary Arteries and Coronary Plaques

In addition to invasive coronary angiography, a considerable number of noninvasive imaging modalities are available that can suggest or demonstrate the presence of coronary artery lesions, either by anatomical or functional or by combined anatomical-functional information showing increased risk of coronary events (Table 2).

**Table 2:** Non-invasive imaging modalities for the study of subclinical coronary artery disease in patients with peripheral artery disease

Imaging modality	Advantages	Drawbacks
Coronary artery calcium score	Low price High availability	Does not visualize non-calcified plaques Low specificity in patients with PAD
Coronary angiography by CT	High sensitivity for stenotic plaque Can image plaque morphology High availability	Ionizing radiation Plaque imaging technically challenging
Cardiac magnetic resonance imaging	Can image plaque morphology High spatial resolution	Technically very challenging Limited availability and high cost
Stress echocardiography	High availability	Does not visualize plaques Operator-dependent
MPI with SPECT	Prognostic information Low spatial resolution	Does not image plaque Ionizing radiation
MPI with PET	Image stress induced ischemia Limited spatial resolution	Does not visualize plaques High cost and low availability Ionizing radiation
FDG-PET/cardiac CT	Combined anatomical and metabolic imaging	High cost and limited availability Ionizing radiation
MPI PET/cardiac CT	Combined anatomical and ischemia imaging	High cost and low availability Ionizing radiation
Specific PET plaque tracer/cardiac CT	Potentially specific identification of vulnerable plaque	Method still at the experimental level Ionizing radiation

MPI=myocardial perfusion imaging, PAD=peripheral artery disease, PET=positron emission tomography, SPECT=single photon emission computed tomography

### Surrogate Measures of Coronary Plaques

Flow-mediated dilatation is a test to measure endothelial dysfunction and thus a surrogate measure of coronary atherosclerosis. The endothelial dysfunction is measured in the brachial artery using ultrasound. Patients with PAD are known to manifest atherosclerosis, and as endothelial dysfunction is an early hallmark of atherosclerosis, it should be expected that flow-mediated dilation would be attenuated in all patients with PAD. This was demonstrated in a study of 44 patients with PAD but without symptoms of coronary artery disease [26]. In addition, the authors found that the degree of flow-mediated dilatation could predict the presence of ischemia on myocardial perfusion imaging. Thus far, these intriguing results have not been validated by other studies.

Coronary artery calcium score (CACS) is calculated from a non-contrast CT of the heart. CACS reflects the total plaque burden and provides incremental prognostic information beyond traditional clinical risk scores (e.g., Framingham and HeartScore) [27]. However, calcification is not a hallmark of vulnerable plaque, and in fact is often seen in stable plaques. CACS, therefore, likely does not image the vulnerable plaque and should be considered a surrogate measure of all plaques, both calcified and non-calcified. Because CACS is a fast, non-invasive, low-cost examination, and subjects the patient to only minimal radiation (typically around 1 mSv), it may potentially serve as a first-line examination in order to select asymptomatic patients with PAD who require subsequent coronary examination. A recent report from the prospective GROUND2 study used this approach in a cohort of 111 patents with PAD and no symptoms of cardiovascular disease [28••]. However, only eight of the patients were found to have 0 CACS and no plaques on coronary CT. As such, CACS as a screening method in patients with PAD would likely add very little information, as most patients will have increased score and will require further imaging.

An intriguing perspective of CACS is the fact that it can reclassify risk in subsets of patients. For example, patients with diabetes and 0 CACS have a cardiovascular event rate similar to patients without diabetes, thereby challenging the notion of diabetes as a coronary heart disease risk equivalent per se [29]. Future studies will need to determine whether this could also apply to patients with PAD.

Another method for imaging calcium is positron emission tomography (PET) using ^18^F-labeled sodium fluoride (NaF). This tracer is deposited by chemisorptions onto hydroxyapatite and is used in oncology to identify sclerotic bone metastasis. Recent evidence suggests that it is not equivalent to CACS imaging [30] but that it can identify “spotty” calcification in the plaques thought to promote plaque vulnerability. In fact, results from one study indicate that NaF PET can identify culprit and ruptured plaques in patients with recent myocardial infarction [31•].

Compared to both NaF and particularly CACS, it may be more attractive to image more specific characteristics of the vulnerable plaque.

### Anatomical Coronary Plaque Imaging

Coronary angiography by CT (CCTA) is a non-invasive method using intravenous contrast, typically to assess luminal stenoses. However, it has been shown that CCTA can also provide images of the vessel wall, and thus plaque characteristics, including positive remodeling, densitometry, and calcification [32]. With improved resolution, even small plaques that are not flow-limiting may potentially be assessed for vulnerability characteristics, although the spatial resolution remains a limitation for direct identification of vulnerable plaques [32]. Another challenge for CCTA is the fact that atherosclerosis is a systemic disease typically involving large parts of the coronary arteries. A comprehensive evaluation of coronary plaque morphology by visual evaluation is a cumbersome task. Multisequence magnetic resonance imaging (MRI) is also able to image the coronary artery wall and plaque morphology [33]., This method is even more technically challenging, however, due to cardiac and respiratory motion of the often small tortuous vessels. MRI does appear to be useful for imaging the larger carotid and aortic plaques.

### Myocardial Ischemia Imaging

There are several methods for examining myocardial perfusion, and thus ischemia. Traditionally, this is accomplished by myocardial perfusion single-photon emission tomography (SPECT) using an intravenous injection of ^99m^technetium-labeled tetrofosmin or sestamibi during stress and rest. The use of PET for perfusion imaging is growing and has several advantages compared to SPECT: higher spatial resolution, faster acquisition, fewer motion artifacts, and absolute flow quantification. The perfusion tracers used for PET imaging are ^82^Rb, ^15^O-labeled water, or ^13^N-labeled ammonia [34]. The primary drawbacks of PET are the limited availability of scanners and perfusion tracers. Both CT and MRI can also image regional myocardial perfusion by dynamic first-pass contrast-enhanced imaging. These methods are less validated in comparison to PET, and particularly SPECT imaging, but the higher resolution is a potential advantage. Dobutamine stress echocardiography combines pharmacological stress with ultrasound imaging to identify left ventricular wall motion abnormalities within the distribution of significant stenotic coronary arteries.

An important advantage of myocardial perfusion imaging is that it is a functional method of imaging perfusion at the myocyte level and thus can determine whether a borderline epicardial stenosis causes a significant change in perfusion. One study (*n* = 111) on the association between PAD and subclinical CAD combined anatomical (CCTA) and ischemia (stress MRI) imaging [28••]. Of 80 patients with plaques on the CCTA (40 with significant and 40 with non-significant stenosis), only 10 were found to have ischemia on subsequent stress MRI.

Perfusion imaging in asymptomatic patients with PAD is useful for identification of areas with silent ischemia, and it is well established that patients without inducible ischemia have very low cardiac event rates (approximately 1% during the following year [35]). However, patients with established PAD will probably have a higher prevalence of non-stenotic vulnerable plaques that cannot be identified with myocardial perfusion imaging, and the negative predictive value of myocardial perfusion imaging in this patient population will likely be lower, as was previously shown in diabetic patients [36].

### Metabolic Imaging of the Vulnerable Plaque

PET imaging of atherosclerosis has thus far focused primarily on fluorine-18-fluorodeoxyglucose (^18^F-FDG). The molecule is a glucose analogue that accumulates in metabolically active cells such as macrophages. The first report on ^18^F-FDG accumulation in the large arteries emerged in 2001 [37], and since then, a large body of evidence has materialized linking FDG uptake to the macrophage contents of high-risk plaques [38–40]. This observation is in line with the emerging consensus of atherosclerosis as an inflammatory disease. A major drawback of imaging coronary atherosclerosis with FDG-PET, however, is the lack of specificity of the tracer, and another limitation is the high uptake of FDG in the myocardium, which produces a suboptimal signal-to-noise ratio. Consequently, in contrast to FDG imaging of carotid plaques, few studies have utilized FDG-PET to image coronary atherosclerosis [41•, 42]. One recent retrospective study indicated a potential future role of arterial FDG-PET in risk stratification of asymptomatic patients. The authors retrospectively identified 513 FDG-PET examinations and revealed a predictive value of arterial FDG uptake for subsequent cardiovascular events in asymptomatic individuals [43].

As an alternative, some researchers have suggested ^18^F-deoxy- mannose (^18^F-FDM) as a more specific tracer than ^18^F-FDG [44], but this needs to be confirmed in clinical trials. To date, no studies have been published on the application of coronary PET imaging in patients with PAD.

### Specific PET Tracers for Plaque Imaging

New and potentially more specific PET tracers for imaging coronary atherosclerosis are emerging [45]. The tracers specifically target the cell-mediated key molecular processes associated with the vulnerable atherosclerotic plaque. The most prominent of these targets include macrophage infiltration, apoptosis, hypoxia, and neoangiogenesis of the intima/media. As discussed, FDG-PET is capable of imaging macrophage infiltration in the plaques but with low specificity. Another potential target is the somatostatin receptor subtype 2 expressed by macrophages. The somatostatin receptor ligand ^68^Ga-DOTATATE has high affinity for somatostatin receptors, and a few studies have suggested a future role in plaque imaging [46–48]. PET tracers for imaging apoptosis, hypoxia, and neoangiogenesis are available, but their use in coronary artery plaque imaging is extremely limited thus far [49•, 50, 51]. A review of new PET and SPECT tracers, as well as tracers used in other modalities, was recently published [52].

### Hybrid Imaging

Hybrid imaging combining anatomical information from a CCTA with metabolic or molecular information from PET imaging may represent a valuable non-invasive modality for the identification of significant microvascular disease and flow-limiting epicardial coronary artery lesions, as well as for demonstration of the characteristics of the vulnerable plaque in selected patients with PAD.

Clinical integrated PET/MRI systems have recently been introduced. MRI can effectively characterize plaque morphology (e.g., hemorrhage and the lipid-rich necrotic core), and the combination with PET-derived molecular imaging holds great potential for in vivo identification of vulnerable plaques. The limited availability of the PET/MRI imaging systems and expensive production of specific PET tracers, however, will likely restrict such a technique to experimental use for some time. Thus far, few reports have been published on hybrid imaging for the study of atherosclerosis [53•], and none have used it for coronary artery imaging. One study combined CACS with CCTA and MRI to identify high-risk PAD patients, but the image selection protocol itself was not validated in the trial [28••].

## Conclusion

An already significant and ever-increasing body of evidence indicates a high prevalence of subclinical coronary atherosclerosis in patients with manifest PAD. The value of general screening of coronary arteries with non-invasive imaging in patients with PAD is not documented. Limited data suggest that imaging patients with PAD before major surgery may be valuable. A palette of potentially specific tracers targeted against plaque vulnerability exists via various imaging modalities including PET, CT, MRI, ultrasound, and imaging combinations. Future studies will determine whether identification of patients with PAD at high risk for CAD complications will eventually improve prognosis.
